# BURSTING THE BUBBLE? CHANGES IN OLDER ADULTS’ LEISURE WITH OTHERS DURING THE PANDEMIC

**DOI:** 10.1093/geroni/igad104.0238

**Published:** 2023-12-21

**Authors:** Amy Rauer, Meagan Stewart, Katherine Fiori, Christina Marini, Jeremy Kanter

**Affiliations:** University of Tennessee, Knoxville, Tennessee, United States; University of Tennessee, Knoxville, Tennessee, United States; Adelphi University, Garden City, New York, United States; Adelphi University, Garden City, New York, United States; Child and Family Studies, Knoxville, Tennessee, United States

## Abstract

After months of social distancing, widespread vaccine availability gave hope to older adults that they could resume social activities. However, as new variants emerged, it was not always clear how safely older adults could connect with others. Thus, the current study examined how older adults’ leisure with others changed as they reintegrated using a sample of 136 older adults in the U.S. (Mage = 68, range: 50-91; 69% females; 93% White; 72% partnered; 61% retired; 88% living independently). Participants reported on leisure at three time points (Summer 2021; Fall 2021; Winter 2022) using a modified version of the Pittsburgh Enjoyable Activities Scale (Pressman et al., 2009). Repeated measures analyses revealed that although there were no differences in the total amount of leisure individuals engaged in over time, with whom they did these activities differed both across time and activities. Participants were most likely to do activities with romantic partners at all three waves, followed by activities alone. Leisure with friends and family was least likely. Although leisure alone and with friends remained stable over time, leisure with a romantic partner and with family declined from T2 to T3, when COVID-19 cases were peaking. Interestingly, declines were seen in both more traditionally social activities (e.g., taking trips, sports) and those usually considered more solitary (e.g., computer use, chores). Despite both rising COVID-19 rates and inconsistent rhetoric on social distancing, older adults were still able to do things they enjoyed, though their ability to do so with loved ones appeared to wane.

